# Association of coagulation markers with the severity of white matter hyperintensities in cerebral small vessel disease

**DOI:** 10.3389/fneur.2024.1331733

**Published:** 2024-02-07

**Authors:** Mingyuan Xu, Jingjing Li, Bu Xu, Qin Zheng, Wenjun Sun

**Affiliations:** ^1^Third Affiliated Hospital, Beijing University of Chinese Medicine, Beijing, China; ^2^Yichang Traditional Chinese Medicine Hospital, Yichang, China

**Keywords:** cerebral small vessel disease, white matter hyperintensities, fibrinogen, coagulation, Mendelian randomization analysis

## Abstract

**Background and purpose:**

This study aimed to explore the correlation and causal relationship between fibrinogen, D-dimer, and the severity of cerebral white matter hyperintensity (MMH).

**Methods:**

A retrospective analysis of 120 patients with cerebral small vessel disease (CSVD) confirmed by head MRI attending the Third Affiliated Hospital of Beijing University of Traditional Chinese Medicine from August 2021 to February 2023 was performed. According to the Fazekas scale score, the patients were divided into 42 cases in the mild group, 44 cases in the moderate group, and 34 cases in the severe group. The levels of fibrinogen and D-dimer were compared among the three groups; the correlations between fibrinogen, D-dimer, and WMH severity were further analyzed; and independent risk factors for WMH severity were explored using the multivariate ordered logistic regression analysis. Furthermore, a two-sample Mendelian randomization (MR) analysis was performed to investigate the genetically predicted effect of fibrinogen and D-dimer on WMH.

**Results:**

As the severity of WMH increased, the levels of D-dimer and fibrinogen also gradually increased, and the results showed a positive correlational association, with significant differences within the groups (all *p* < 0.05); the multivariate ordered logistic regression model showed that after adjusting for the relevant covariates, D-dimer (OR = 5.998, 95% CI 2.213–16.252, *p* < 0.001) and fibrinogen (OR = 9.074, 95% CI 4.054–20.311, *p* < 0.001) remained independent risk factors for the severity of WMH. In the MR study, the random-effect inverse variance weighted (IVW) model showed that increased levels of genetically predicted D-dimer (OR, 1.01; 95% confidence interval 0.95–1.06; *p* = 0.81) and fibrinogen (OR, 1.91; 95% confidence interval 0.97–3.78; *p* = 0.06) were not associated with increased risk of WMH. The authors did not obtain strong evidence of a direct causal relationship between D-dimer, fibrinogen, and WMH.

**Conclusion:**

In this retrospective-based study, the authors found possible associations between D-dimer, fibrinogen, and WMH, but there was no obvious causal evidence. Further efforts are still needed to investigate the pathophysiology between D-dimer, fibrinogen, and WMH.

## 1 Introduction

Cerebral small vessel disease (CSVD) is a very common neurological disorder, and cerebral white matter hyperintensity (WMH) is the most common imaging feature of CSVD (1) and is also an important risk factor for cognitive impairment and dementia (2–4). Coagulation mechanisms play a critical role in the pathophysiology of WMH. Coagulation mechanisms are thought to be closely associated with vascular endothelial function, blood–brain barrier permeability, and neurovascular dysfunction, which may influence the development of WMH (5). Studies have shown a close association between alterations in coagulation biomarkers retinoic acid receptor responder 2, von Willebrand factor, and WMH (6).

To the best of our knowledge, the correlations of D-dimer and fibrinogen with WMH have not been adequately investigated. Previous studies have shown that cerebral autosomal dominant arteriopathy with subcortical infarcts and leukoencephalopathy (CADASIL) patients and sporadic CSVD patients showed significantly higher plasma fibrinogen levels than healthy controls, and there is a correlation between high levels of plasma fibrinogen and the severity of WMH in CADASIL patients (7). A single-center cohort study showed that fibrinogen and D-dimer were correlated with the burden of radiological markers of CSVD (8). Nevertheless, the relationships of D-dimer and fibrinogen with the presence and severity of WMH remain unknown.

In this study, the authors evaluated the associations of D-dimer and fibrinogen with WMH in CSVD, in a retrospective analysis. Furthermore, the authors conducted Mendelian randomization (MR) analysis, which can avoid potential unmeasured confounders and reverse causation by using genetic variants as instrumental variables, to verify the causal association of the association between D-dimer, fibrinogen, and WMH.

## 2 Materials and methods

### 2.1 Participants

A total of 120 patients with WMH in CSVD who were admitted to the Third Affiliated Hospital of Beijing University of Traditional Chinese Medicine from August 2021 to February 2023 were retrospectively examined.

### 2.2 Inclusion criteria

① Patients who met the “Chinese Consensus on Diagnosis and Therapy of Cerebral Small Vessel Disease 2021” CSVD diagnostic criteria (9); ② patients aged between 18 and 90 years; ③ patients with head MRI image data available for analysis; and ④ patients with no communication or comprehension disorders.

### 2.3 Exclusion criteria

Patients who ① had suffered from large intracranial vessels stenosis or occlusion (stenosis degree ≥50%) as indicated by MRA; ② had suffered from large cervical vessels stenosis or occlusion as indicated by ultrasound of cervical blood vessels; ③ had suffered from cerebral white matter degeneration or cardiac cerebral infarction due to non-vascular factors (monoxide encephalopathy, multiple sclerosis, metabolism, toxicity, and infections); ④ had other central nervous system (CNS) diseases, such as cerebral hemorrhage, cerebral infarction, epilepsy, multiple sclerosis, intracranial tumors, cranial and cerebral trauma, and poisoning; and ⑤ had a history of severe organ failure or malignant tumors. The retrospective study was conducted in accordance with the principles of the Declaration of Helsinki and was approved by the Ethics Committee of the Third Affiliated Hospital of Beijing University of Chinese Medicine (institutional review board [IRB] approval number: ECSL-BZYSY-2022-04).

### 2.4 Assessment of the severity of WMH and grouping

All patients completed 1.5 T or 3.0 T head MRI, which mainly included T1-weighted imaging (T1WI), T2-weighted imaging (T2WI), fluid-attenuated inversion recovery (FLAIR), and diffusion-weighted imaging (DWI) sequences. WMH was defined as the areas of intensity signal on the T2-weighted FLAIR sequence that are divided into periventricular WMH (PVWMH) and deep WMH (DWMH) (10). The severity of WMH on cranial MRI was graded using the Fazekas scale: (1) PVWMH: presenting a cap-like or pencil-like thin layer lesion, 1 point; presenting a smooth halo, 2 points; presenting irregular PVWMH even extending into the deep white matter of the brain, 3 points; (2) DWMH: presenting a punctate lesion, 1 point; beginning confluence of lesion, 2 points; and merging in a large area of the lesion, 3 points. The scores of the two parts were added together to form a total score, indicating the severity of WMH (2). The imaging assessment of each WMH was rated by two well-trained neurologic imaging physicians who were blinded to the participant’s clinical data, and images with inconsistent results were finally assessed by another senior neurologist who was blinded to the initial results. Finally, a total score of 1 to 2 was categorized as mild group, 3 to 4 as moderate group, and 5 to 6 as severe group.

### 2.5 Data collection

We collected patient demographics (age and sex), risk factors (smoking history and alcohol consumption), medical history (hypertension, diabetes, and hyperlipidemia), laboratory indexes including alkaline phosphatase (ALP), γ-glutamyltransferase (GGT), uric acid (UA), β2-microglobulin (β2-M), D-dimer, and fibrinogen. Fasting venous blood samples were collected at baseline. All patient data were collected from the electronic medical record system of the hospital.

### 2.6 MR study design

**Figure 1:**
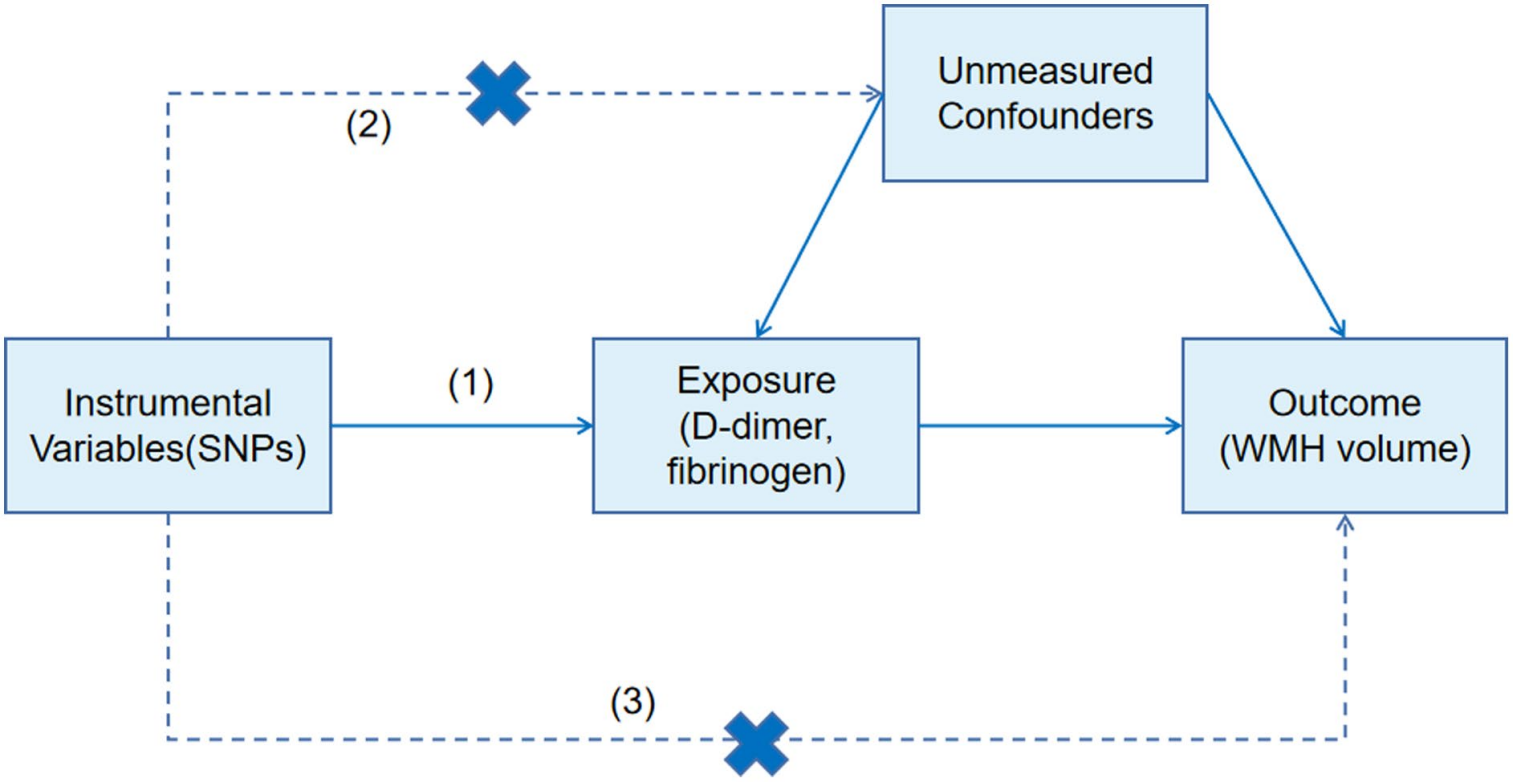
Mendelian randomization design and main hypotheses.

We further designed a two-sample MR approach to estimate the effect of fibrinogen and D-dimer on WMH. MR analysis can be used for unbiased detection of causal effects of risk factors on diseases because the genetic variations are allocated randomly at conception (11). The genetic variants used in an MR design must meet three assumptions: (1) the genetic variants used as instrumental variables are strongly associated with the risk factor of interest (D-dimer, fibrinogen); (2) the genetic variants should not be associated with other confounders; and (3) the genetic variants are associated with the disease (WMH) only through the investigated risk factors (D-dimer, fibrinogen) (12) (Figure 1). The authors obtained the latest published summary-level GWAS data for WMH from the Cerebrovascular Disease Knowledge Portal (13).^1^ The GWAS summary statistics data of D-dimer were obtained from the genomic atlas of plasma proteome of 3,301 individuals (14), and the GWAS summary statistics data of fibrinogen were obtained from genome-wide analysis of biomarkers of 9,762 individuals (15). Appropriate ethical approval and patient-informed consent were obtained from the original studies. Analyses of all phenotypes were based on subjects of European ancestry.

Single nucleotide polymorphisms (SNPs) associated with D-dimer and fibrinogen were selected as instrumental variables (IVs), which reached genome-wide significant levels (*p* < 5 × 10^−6^). The authors removed SNPs with linkage disequilibrium based on the *r*^2^ < 0.01 linkage disequilibrium threshold within 1,000 kb as instrumental variables for the final MR study to avoid bias caused by relevant instrumental variables. The inverse-variance weighted (IVW) method was used to calculate the odds ratio (OR) and 95% confidence interval as the main method to estimate the association of genetically predicted D-dimer and fibrinogen with WMH. MR-Egger and weighted median methods were used as supplementary methods to IVW (16). In the sensitivity analysis, the authors used the MR-Egger intercept test and MR-PRESSO (Pleiotropy Residual Sum and Outlier) to assess horizontal pleiotropy. The mr_heterogeneity test was used to assess heterogeneity. All analyses were conducted by the “TwoSampleMR” package in version R 4.2.3, and a *p*-value <0.05 was considered statistically significant.

### 2.7 Statistical analysis

Statistical analysis was performed using SPSS 25.0 software, and normality was tested using the Shapiro–Wilk test. Continuous variables conforming to normal distribution were expressed as mean ± standard deviation ( $\bar{x}$ ± s). The ANOVA test was used for comparison between multiple groups, and if the difference between groups was statistically significant, the LSD method was further used to compare with one another. Categorical variables were summarized as count (percentage) [*n* (%)] and compared using the chi-square test. Categorical variables were analyzed using Spearman correlation analysis, and continuous variables were analyzed by Pearson correlation analysis. The independent influencing factors of the degree of WMH damage were analyzed by a multivariate ordered logistic regression model, and a *p*-value <0.05 was considered statistically significant.

## 3 Results

### 3.1 Baseline characterization

**Figure 2:**
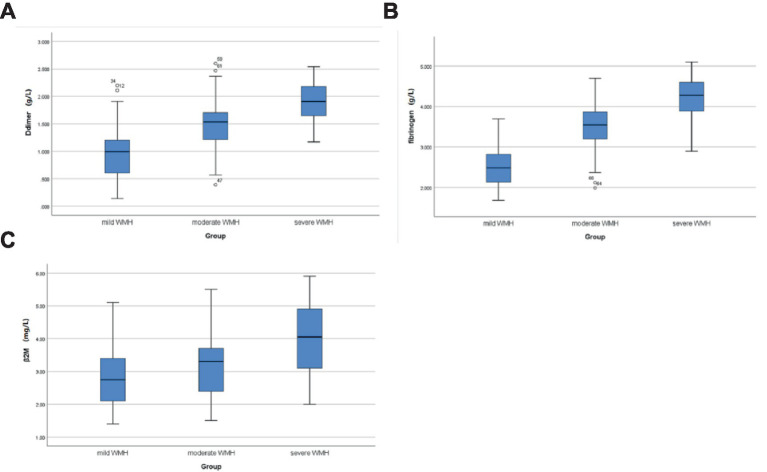
Comparison of D-dimer, fibrinogen, and β2-M levels in CSVD patients with different WMH severity. **(A)** Comparison of D-dimer levels in the mild, moderate, and severe groups; **(B)** comparison of fibrinogen levels in the mild, moderate, and severe groups; and **(C)** comparison of β2M levels in the mild, moderate, and severe groups.

A total of 120 patients with CSVD were included, 54 (45%) individuals were men and 66 (55%) were women; the age range was 50–89 years, with a mean of (71.1 ± 9.3) years. There was no statistically significant difference between the mild group in terms of sex, smoking, alcohol consumption, diabetes mellitus, hyperlipidemia, ALP, GGT, and UA compared with the moderate and severe groups (*p* > 0.05); as the severity of WMH increased, the age, β2-M, D-dimer, and fibrinogen levels of the three groups increased gradually, and there exist statistically significant differences among the three groups (all *p* < 0.05). A history of hypertension was also a risk factor for the severity of WMH. The severe group had the highest proportion of people suffering from hypertension, 82.4% (28 cases), followed by the moderate group, 75% (33 cases), and finally the mild group, 54.8% (23 cases), with statistically significant differences (*p* < 0.05) (Table 1; Figure 2).

**Table 1:** Comparison of baseline information of three groups of CSVD patients.

Variable	Group			F/x^2^	*p-*value
	Mild group (*n* = 42)	Moderate group (*n* = 44)	Severe group (*n* = 34)		
Sex				1.612	0.447
Male (*n*,%)	22 (52.4)	19 (43.2)	13 (38.2)		
Female (*n*,%)	20 (47.6)	25 (56.8)	21 (61.8)		
Age ( $\bar{x}$ ± s, years)	68.29 ± 7.10	70.52 ± 10.059	75.32 ± 9.322	6.009	0.003*
Personal History
Smoking (*n*,%)	5 (11.9)	12 (27.3)	10 (29.4)	4.210	0.122
Drinking (*n*,%)	6 (14.3)	13 (29.5)	8 (23.5)	2.898	0.235
Past medical history
Hypertension (*n*,%)	23 (54.8)	33 (75.0)	28 (82.4)	7.638	0.022*
Diabetes (*n*,%)	16 (38.1)	18 (40.9)	17 (50.0)	1.162	0.559
Hyperlipidemia (*n*,%)	26 (61.9)	28 (63.6)	20 (58.8)	0.189	0.910
Serum markers
ALP ( $\bar{x}$ ± s, U/L)	77.190 ± 25.437	83.409 ± 28.299	84.176 ± 29.221	0.777	0.462
GGT ( $\bar{x}$ ± s, U/L)	42.000 ± 20.527	39.227 ± 17.608	42.265 ± 17.686	0.335	0.716
UA ( $\bar{x}$ ± s, umol/L)	313.738 ± 98.898	331.409 ± 97.376	310.176 ± 82.010	0.601	0.550
β2-M ( $\bar{x}$ ± s, mg/L)	2.850 ± 0.958	3.180 ± 0.960	4.090 ± 1.128	14.898	*p*<0.001**
D-dimer ( $\bar{x}$ ± s, mg/L)	1.004 ± 0.498	1.452 ± 0.470	1.889 ± 0.388	35.117	*p*<0.001**
Fibrinogen ( $\bar{x}$ ± s, g/L)	2.530 ± 0.515	3.474 ± 0.566	4.190 ± 0.598	84.728	*p*<0.001**

ALP, alkaline phosphatase (ALP); GGT, γ-glutamyltransferase (GGT); UA, uric acid (UA); β2-M, β2-microglobulin. ^*^Between-group differences *p* < 0.05. ^**^Between-group differences *p* < 0.001.

### 3.2 Spearman and Pearson correlation analysis

Spearman correlation analysis of age, hypertension, β2-M, D-dimer, and fibrinogen with WMH was performed. As shown in Table 2, age (*r* = 0.275, *p* = 0.002), history of hypertension (*r* = 0.239, *p* = 0.009), β2-M (*r* = 0.474, *p* < 0.001), D-dimer (*r* = 0.656, *p* < 0.001), and fibrinogen (*r* = 0.797, *p* < 0.001) were all positively correlated with the severity of WMH. Pearson correlation analysis was performed for age, β2-M, D-dimer, and fibrinogen. Continuous variables met the normality test (*p* > 0.05). As shown in Table 3, there was a positive correlation between fibrinogen and age; and β2-M, D-dimer, and fibrinogen were all positively correlated with each other (all *p* < 0.05). Spearman correlation analysis of age, β2-M, D-dimer, and fibrinogen with hypertension was performed. As shown in Table 4, there was a correlation between fibrinogen and history of hypertension (*p* < 0.05) (Tables 2–4).

**Table 2:** Spearman correlation analysis.

Variable		Age	Hypertension	β2-M	D-dimer	fibrinogen
WMH score	*r*-value	0.275	0.239	0.474	0.656	0.797
	*p-*value	0.002^*^	0.009^*^	<0.001^**^	<0.001^**^	<0.001^**^

β2-M, β2-microglobulin.^*^Between-group differences *p* < 0.05. ^**^Between-group differences *p* < 0.001.

**Table 3:** Pearson correlation analysis.

Variable		β2-M	D-Dimer	Fibrinogen
Age	*r*-value/*p*-value	0.151/0.100	0.119/0.194	0.213/0.019^*^
β2-M	*r*-value/*p*-value		0.282/0.002^*^	0.432/<0.001^**^
D-dimer	*r*-value/*p*-value			0.572/<0.001^**^

β2-M, β2-microglobulin.^*^Between-group differences *p* < 0.05. ^**^Between-group differences *p* < 0.001.

**Table 4:** Spearman correlation analysis.

Variable		Age	β2-M	D-dimer	Fibrinogen
Hypertension	*r*-value	0.156	0.087	−0.016	0.202
	*p*-value	0.088	0.344	0.864	0.027^*^

β2-M, β2-microglobulin. ^*^Between groups differences *p* < 0.05.

### 3.3 Multivariate ordered logistic regression analysis

WMH severity was used as the dependent variable (X = 1, mild; X = 2, moderate; and X = 3, severe), hypertension as the factor (X = 0, absent; X = 1, present), and age, β2-M, D-dimer, and fibrinogen as covariates were included in the multivariate ordered logistic regression model. Our logistic regression model passed the parallel line test (*p* = 0.499) and satisfied the “proportional odds” assumption. The results showed that after adjusting for the relevant covariates, D-dimer (OR = 5.998, 95% CI 2.213–16.252, *p* < 0.001) and fibrinogen (OR = 9.074, 95% CI 4.054–20.311, *p* < 0.001) remained independent risk factors for the severity of WMH. An increase of 1 mg/L D-dimer suggested a 4.9 times increased risk of WMH severity, and an increase of 1 g/L fibrinogen suggested an 8.1 times increased risk of WMH severity (Table 5).

**Table 5:** Multivariate ordered logistic regression analysis of the degree of WMH.

Variable	Β	SE	Wald	Df	*p*-value	OR	95%CI	
							Lower limit	Upper limit
Age	0.043	0.025	3.069	1	0.080	1.044	0.995	1.096
Hypertension	−0.975	0.518	3.535	1	0.060	0.377	0.137	1.042
β2-M	0.428	0.222	3.712	1	0.054	1.534	0.993	2.372
D-dimer	1.791	0.509	12.406	1	<0.001^**^	5.998	2.213	16.252
Fibrinogen	2.205	0.411	28.781	1	<0.001^**^	9.074	4.054	20.311

β2-M, β2-microglobulin; OR, odds ratio; CI, confidence interval. ^*^Between-group differences *p* < 0.05. ^**^Between-group differences *p* < 0.001.

### 3.4 Two-sample Mendelian studies

**Figure 3:**
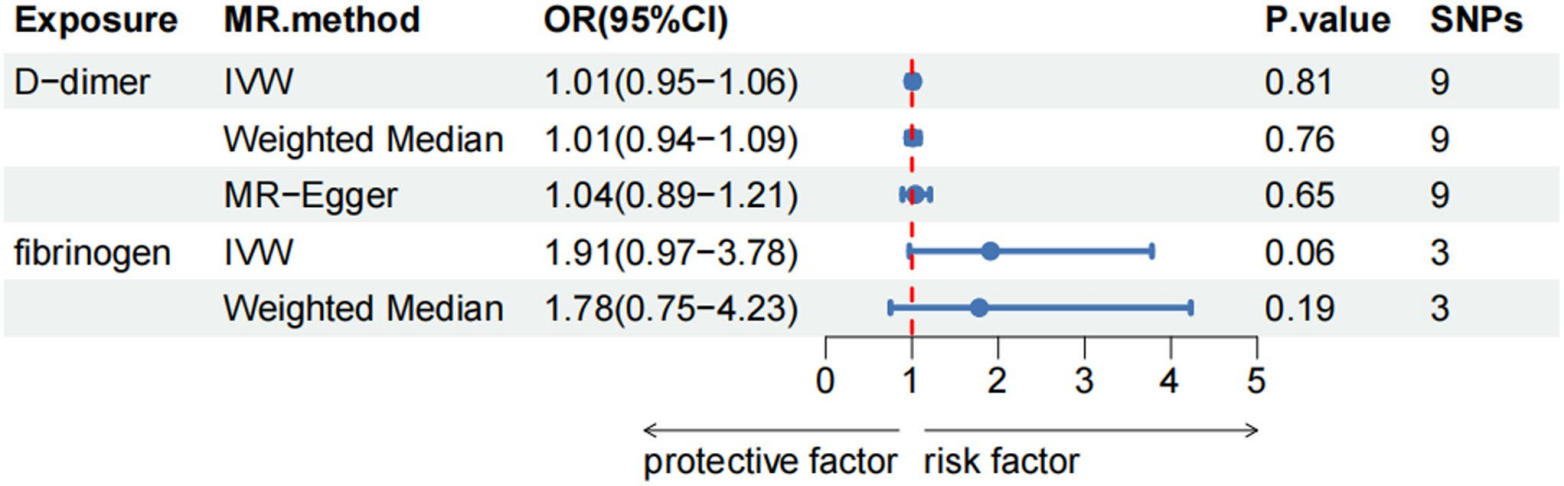
Results of the Mendelian randomization analysis for the effect of the D-dimer and fibrinogen concentration on WMH.

**Figure 4:**
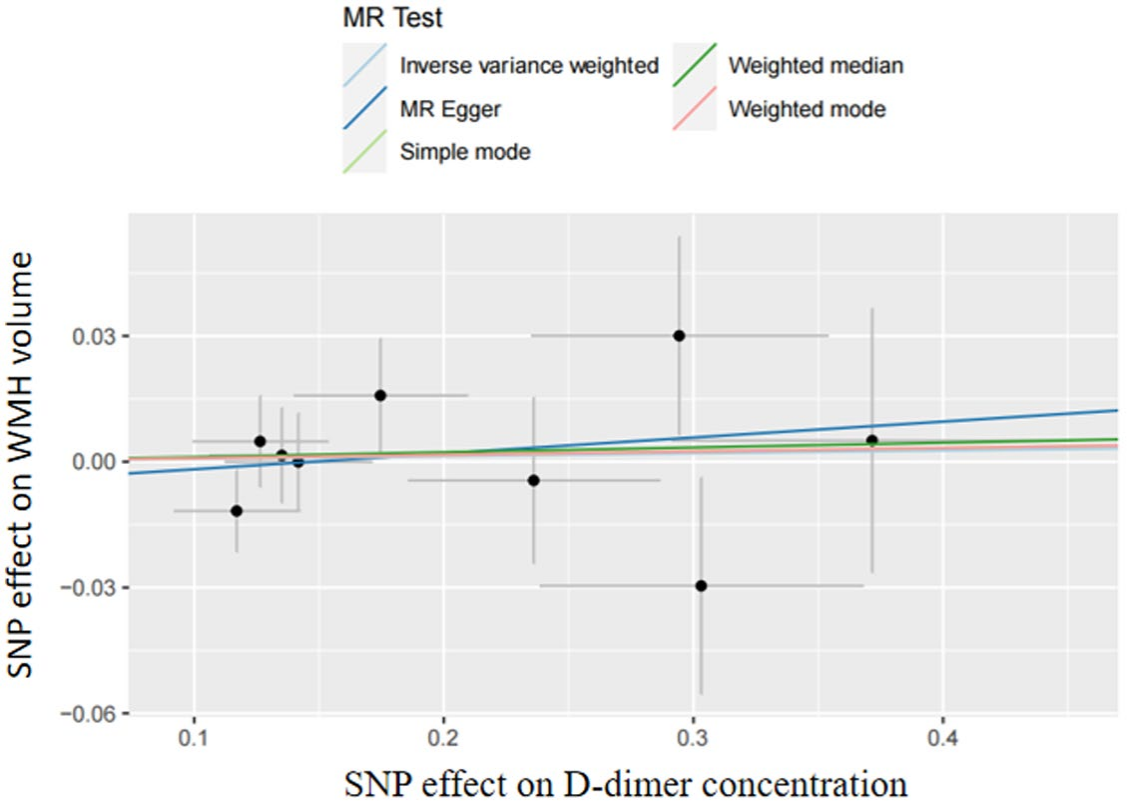
Mendelian randomization analysis of the effect of D-dimer on WMH.

**Figure 5:**
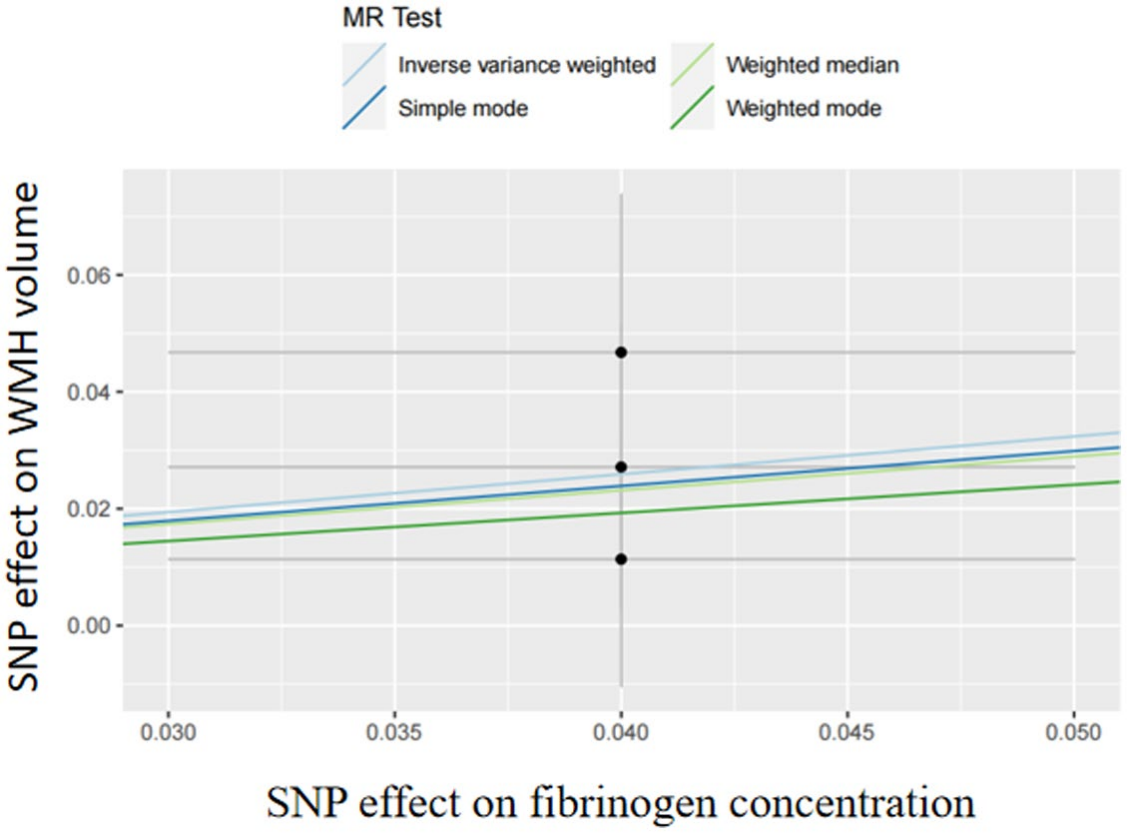
Mendelian randomization analysis of the effect of fibrinogen on WMH.

In two-sample MR analyses, the authors identified nine and three SNPs as IVs in our MR analyses of D-dimer and fibrinogen on WMH, respectively. The random-effects IVW models showed that genetically predicted increased levels of D-dimer (OR,1.01; 95% CI 0.95–1.06; *p* = 0.81) and fibrinogen (OR,1.91; 95% CI 0.97–3.78; *p* = 0.06) were not associated with increased WMH volume. The authors did not obtain strong evidence of a direct causal relationship between genetically mediated coagulation markers (D-dimer and fibrinogen) and WMH. The MR-Egger and weighted-median methods were consistent with the results of the IVW analyses. There was no multiplicity and heterogeneity in sensitivity analyses for both D-dimer and fibrinogen (all *p* > 0.05). The MR-Egger analysis did not show any genetic correlation between fibrinogen concentration and WMH, probably because the number of exposed SNPs in fibrinogen is low (Figures 3–5; Table 6).

**Table 6:** SNPs in MR studies.

MR study	SNP
D-dimer-WMH	rs144846383	rs150224075	rs16850834	rs1891118	rs28399331	rs59234924	rs608406	rs6556833	rs9404903
Fibrinogen-WMH	rs113688763	rs77048498	rs78965196

## 4 Discussion

The authors examined associations between coagulation markers and WMH using retrospective analysis and complementary MR analyses. Our retrospective analysis showed that elevated D-dimer and fibrinogen levels suggested a correlation with WMH severity. After adjusting for associated risk factors, the multivariate ordered logistic regression model showed that elevated D-dimer and fibrinogen levels were independent risk factors for WMH severity, independent of traditional vascular risk factors. However, in two-sample MR analyses, there was no strong evidence of a causal effect of D-dimer, fibrinogen, and WMH. The authors consider that D-dimer and fibrinogen may indirectly contribute to the development of WMH through other mechanisms. To the best of our knowledge, this is the first MR study on the relationship between WMH and coagulation markers.

The pathological mechanisms of WMH mainly include insufficient cerebral perfusion, blood–brain barrier damage, and endothelial cerebrovascular dysfunction (17, 18). Coagulation mechanisms play a critical role in this. Our results are consistent with previous studies that a cluster of plasma markers reflecting coagulation correlated with WMH (6). On the one hand, coagulation abnormalities are the basis of endothelial dysfunction, causing damage to endothelial cells and thus disrupting the integrity of the blood–brain barrier (19). On the other hand, hypercoagulable states reduce cerebral blood flow, leading to microcirculatory disturbances that promote the progression of WMH (20).

Fibrinogen, as coagulation factor I in the coagulation system, is an indicator of the hypercoagulable state of the blood. High concentration of fibrinogen leads to excessive blood viscosity and increases platelet aggregation and the release of endothelial cell adhesion molecules. Then, it induces the formation of thrombosis in small arteries and veins and promotes small arterial sclerosis; at the same time, excessive blood viscosity leads to a decrease in cerebral blood flow (21). Insufficient blood flow to the cerebral white matter terminals, secondary to microcirculatory disturbances, causes diffuse demyelinating changes in the cerebral white matter, and the development of long-term chronic cerebral ischemia exacerbates WMH. Previous studies have shown that cerebral blood flow is lower in the periventricular NAWM in patients with ischemic WMH compared with controls (22). Low cerebral blood flow in the NAWM correlates strongly with new-onset WMH (20). A postmortem study confirmed a significant correlation between fibrinogen and microvascular stenosis in the cerebral white matter. The levels of fibrinogen were significantly higher in patients with PVWMH who had an infarction than patients without PVWMH. Periventricular white matter, as a watershed, is highly susceptible to hypoperfusion due to small artery stenosis. Chronic hypoperfusion caused by microvascular stenosis leads to blood–brain barrier dysfunction through loss of pericytes and impairment of endothelial integrity, which results in extravasation of fibrinogen and promotes the accumulation of microglia. At the same time, microvascular stenosis results in a reduction of cerebral blood flow, leading to chronic ischemia of cerebral white matter, which ultimately results in demyelination of cerebral white matter (23). Studies revealed that increased blood–brain barrier damage was correlated with decreased cerebral blood flow. This relationship is strongest in the WMH and its vicinity and is less pronounced at greater distances (24). A 2-year follow-up study showed that fibrinogen was significantly associated with the risk of new lacunes or white matter lesions progression, supporting the role of coagulation factors in CSVD imaging progression (25).

D-dimer as a biomarker reflecting coagulation also plays an important role in the coagulation mechanism. D-dimer is a plasmin-derived soluble degradation product of cross-linked fibrin, produced under the action of coagulation factor XIIIa, mainly reflecting the function of fibrinolysis. D-dimer can be used as a specific molecular marker of hypercoagulable states and hyperfibrinolysis, which is of great value in the diagnosis of thrombosis (26). Consistent with previous studies, a retrospective analysis showed that D-dimer levels were significantly higher in the CSVD group than in the healthy control group and significantly higher in the severe WMH group than in the mild group (27). High levels of D-dimer indicate a hypercoagulable state of the blood, which promotes vascular thrombosis, then increases the risk of small vessel occlusion, and leads to microcirculatory disturbances. The ARIC study found a positive correlation between D-dimer and silent brain infarcts (28). A single-center cohort study showed a correlation between elevated D-dimer and WMH, with elevated fibrinogen and D-dimer, suggesting a prethrombotic profile. The low inflammatory state associated with this feature is related to the degree of WMH (8).

Components of the coagulation system can also drive CNS inflammation leading to WMH. Fibrinogen is not only an indicator of hypercoagulable states, but also a biomarker of the inflammatory response (29). When the blood–brain barrier is disrupted, fibrinogen enters the CNS and is rapidly converted to fibrin, which promotes microglia activation and generates reactive oxygen species and nitric oxide. It further promotes endothelial cell and neuronal damage leading to endothelial dysfunction (30). At the same time, the release of inflammatory mediators, such as TNF-α and IL-6, which exacerbate local oxidative stress and lead to inflammatory response and neurodegeneration (31). Studies have shown that the number of periventricular and subcortical microglia is significantly positively correlated with fibrinogen (23). Previous studies have also demonstrated that CNS inflammation is associated with the inward flow of fibrinogen through the dysfunctional blood–brain barrier and the activation of the intrathecal coagulation cascade (32). Chronic cerebral ischemia and low perfusion environments provide conditions for neurological inflammatory responses. Neurological immune inflammation leads to disruption of the blood–brain barrier by mediating endothelial dysfunction, which contributes to disruption of the neurovascular unit and induces WMH changes.

### 4.1 Limitations

This study analyzed the relationship between fibrinogen, D-dimer, and the severity of WMH in CSVD, which has some limitations. First, this is a small-sample retrospective study, and clinical trials with larger sample sizes are needed to explore the relationship between coagulation markers and WMH in depth. Second, in the retrospective and MR studies, the authors focused on a subset of coagulation markers (fibrinogen and D-dimer), and future studies should examine other coagulation markers. Third, in the observational analyses, our results showed that there was a positive correlation between D-dimer, fibrinogen, and the severity of WMH, but unavoidable residual confounders may still exist. Meanwhile, in MR analysis, there was not found obvious causal relationship. Fourth, this article focused only on WMH, without considering all the other features of CSVD. Finally, in MR analyses, the population was limited to individuals of European ancestry and further genome-wide correlation studies of CSVD in East Asian ethnic groups should be performed to confirm the correlation between coagulation markers and WMH.

## 5 Conclusion

In conclusion, our study showed that there was a positive correlation association between D-dimer, fibrinogen, and the severity of WMH. Moreover, D-dimer and fibrinogen were independent risk factors for the severity of WMH in patients with CSVD. However, in the MR analyses, there was no significant causal relationship. A larger GWAS is needed to investigate in depth the relationship between other coagulation markers and WMH, and a larger sample size is needed for the experimental studies to further explore causality.

## Data availability statement

The original contributions presented in the study are included in the article/supplementary materials, further inquiries can be directed to the corresponding authors.

## Ethics statement

The studies involving humans were approved by Ethics Committee of the Third Affiliated Hospital of Beijing University of Chinese Medicine. The studies were conducted in accordance with the local legislation and institutional requirements. The participants provided their written informed consent to participate in this study.

## Author contributions

MX: Visualization, Writing – original draft, Writing – review & editing. JL: Data curation, Formal analysis, Investigation, Writing – review & editing. BX: Formal analysis, Investigation, Writing – review & editing. QZ: Conceptualization, Methodology, Project administration, Writing – review & editing. WS: Conceptualization, Project administration, Resources, Supervision, Writing – review & editing.

## Footnotes

^1^https://www.cerebrovascularportal.org
